# Photocatalytic degradation of methylene blue under visible light by cobalt ferrite nanoparticles/graphene quantum dots

**DOI:** 10.3762/bjnano.15.43

**Published:** 2024-04-29

**Authors:** Vo Chau Ngoc Anh, Le Thi Thanh Nhi, Le Thi Kim Dung, Dang Thi Ngoc Hoa, Nguyen Truong Son, Nguyen Thi Thao Uyen, Nguyen Ngoc Uyen Thu, Le Van Thanh Son, Le Trung Hieu, Tran Ngoc Tuyen, Dinh Quang Khieu

**Affiliations:** 1 University of Pharmacy and Medicine, Hue University, Vietnam; 2 University of Sciences, Hue University, Vietnam; 3 Center for Advanced Chemistry, Institute of Research & Development, Duy Tan University, Vietnam; 4 Faculty of Natural Sciences, Duy Tan University, Vietnam; 5 University of Education and Science, The University of Danang, Vietnam

**Keywords:** cobalt ferrite, graphene quantum dots, methylene blue

## Abstract

A simple approach was developed to synthesize cobalt ferrite nanoparticles/graphene quantum dots (CF/GQDs). The material was prepared from a homogeneous mixture of iron nitrate, cobalt nitrate, and starch at 140, 180 and 200 °C in a 24 h thermal hydrolysis process. The obtained materials were characterised by using X-ray diffraction, scanning electron microscopy, transmission electron microscopy, ultraviolet–visible diffuse reflectance spectroscopy, Fourier-transform infrared spectroscopy, photoluminescence spectroscopy, vibrating-sample magnetometry, and nitrogen adsorption/desorption isotherms. Cobalt ferrite crystals of around 8–10 nm and graphene quantum dots formed directly at 200 °C. Stacking GQDs sheets onto the CF nanoparticles resulted in CF/GQDs nanoparticles. The nanocomposite exhibits satisfactory fluorescent and superparamagnetic properties, which are vital for catalytic applications. The CF/GQDs catalyse significantly the degradation of methylene blue (MB) under visible light. The catalyst can be recycled with an external magnetic field and displays suitable stability. Also, it was reused in three successive experiments with a loss of efficiency of about 5%. The CF/GQDs are considered as an efficient photocatalyst for MB degradation and other dyes.

## Introduction

Graphene quantum dots (GQDs) have unique properties, including photoluminescence, biocompatibility, slight chemical toxicity, inertness, and excellent photostability [1–2]. Graphene quantum dots consist of graphene sheets that are single-, double-, or multilayered, all smaller than 10 nm in thickness and 100 nm in width. Therefore, GQDs possess an ultralarge specific surface area thanks to nanometre-sized graphene sheets. Graphene quantum dots are fabricated via two techniques, that is, by breaking graphene sheets or from molecules with aromatic structure (fullerenes, starch, and carbohydrates) [3]. However, often GQDs are only stable in solvents, which limits their application in fields that require their solid form, such as in adsorption and photocatalytic, or electrochemical applications [4].

Several magnetic nanomaterials that can be recycled and reused have been developed for catalysis or adsorption [5]. Among them are ferrites with the general formula of MFe_2_O_4_ (M: Mn, Fe, Co, Ni, Cu, and Zn). They are important magnetic materials. Among the spinel ferrites, CoFe_2_O_4_ (CF) is one of the most important soft-magnetic ferrite materials because of its ferromagnetic properties, high magnetic and thermal stability, low conductivity, and anisotropy [6]. The controllable synthesis of spherical CF nanoparticles from corresponding salts and Arabic gum as surfactant agent using a hydrothermal method was reported [7]. The effect of oleylamine concentration on the physicochemical properties of CF synthesized via a solvothermal process has been presented [8]. However, ferrites exhibit self-agglomeration due to their magnetic nature. Combining different functional materials to prepare multiple-component photocatalysts is a common way to overcome the issues of single-phase photocatalysts [9].

Stacking GQDs sheets onto CF nanoparticles not only prevents CF from agglomeration but also forms heterojunction contacts, which possibly reduce the recombination of the photo-induced electron–hole pairs in photocatalytic processes. Ramachandran et al. reported the synthesis of a CF/GQDs nanocomposite by co-precipitating cobalt ferrite nanoparticles on graphene quantum dots prepared from citric acid [10]. Naghshbandi et al. synthesized CF/GQDs from a mixture of GQDs, prepared by carbonising citric acid in a controlled manner, and CF; the material was used to reduce 4-nitrophenol with a very high rate constant compared to the individual components [11].

Despite various advancements in preparation techniques for nanocatalysts, the development of a well-controlled method remains a challenge. In this study, we describe an eco-friendly facile hydrothermal method to prepare magnetic CF/GQDs nanocomposites. The CF nanoparticles, 8–10 nm in diameter, were highly dispersed in a graphene quantum dot matrix and directly formed at 200 °C. Stacking GQDs sheets onto the CF nanoparticles resulted in CF/GQDs nanoparticles. Magnetic properties, morphology, structure, and fluorescence of the nanocomposites were studied, and the photocatalytic degradation of methylene blue as a dye model and the mechanism of methylene degradation were also addressed.

## Experimental

### Materials

Cobalt(II) nitrate hexahydrate (Co(NO_3_)_2_·6H_2_O, 98%), iron(III) nitrate nonahydrate (Fe(NO_3_)_3_·9H_2_O, 98%), and starch ((C_6_H_10_O_5_)*_n_*, 98%) were purchased from Merck, Germany. Methylene blue trihydrate (C_16_H_18_ClN_3_S·3H_2_O), isopropanol (C_3_H_8_O), potassium iodide (KI), and potassium bromate (KBrO_3_) were also purchased from Merck, and benzoquinone (C_6_H_4_O_2_) was provided by Sigma-Aldrich, USA. Sodium hydroxide (NaOH, ≥96%) and hydrogen chloride (HCl, 38%) were purchased from Xilong, China.

### Instruments

X-ray diffraction (XRD) patterns were recorded by using a D8 Advance (Bruker, Germany) with Cu Kα radiation (λ = 0.154 nm). Fourier-transform infrared (FTIR) spectra were recorded on a IR-Prestige-21 (Shimadzu). Raman spectra were recorded on an Xplora Plus instrument (Horiba, Japan) in a frequency range from 200 to 2000 cm^−1^ and with an excitation wavelength of 785 nm. The nitrogen adsorption/desorption isotherms were recorded by using a Tristar-3030 system (Micromeritics, USA). The magnetic properties were measured on a Micro Sense vibrating-sample magnetometer (VSM) at room temperature. Scanning electron microscopy (SEM) observations and energy-dispersive X-ray elemental mapping (EDX mapping) were carried out on a Hitachi S-4800 FESEM (Japan). High-resolution transmission electron microscopy (HR-TEM) observation was performed with a JEM 1010. The intermediates in the MB degradation were determined by using an Agilent 1100 LC/MS-MS system with an electron spray ionization source combined with an ion trap.

### Synthesis of CoFe_2_O_4_, CoFe_2_O_4_/GQDs, and GQDs

Cobalt ferrite was synthesized through a thermal hydrolysis method according to the literature [12]. Briefly, mixtures of Co(NO_3_)_2_·6H_2_O, Fe(NO_3_)_3_·9H_2_O, and starch (C_6_H_10_O_5_)*_n_* with different compositions were prepared (Table 1). Each mixture was heated to 80–85 °C for 1 h in a flask to hydrolyse the starch. Then, the mixture was transferred to a 100 mL Teflon-lined autoclave and heated at 200 °C for 24 h. After hydrolysis, the product was cooled to room temperature. The brown solid was centrifuged, washed several times with distilled water, and calcinated at 700 °C for 3 h to obtain cobalt ferrite. The products were denoted as CF0.5, CF0.67, CF1.0, and CF2.0, where the numeral is the initial Fe/Co molar ratio.

**Table 1:** Precursor compositions for cobalt ferrite samples from different Fe/Co molar ratios.

	Co(NO_3_)_2_·6H_2_O (g)	Fe(NO_3_)_3_·9H_2_O (g)	(C_6_H_10_O_5_)*_n_* (g)	H_2_O (g)

CF0.5	4.95	3.44	6.20	82.7
CF0.67	3.71	3.44	5.17	68.9
CF1.0	2.48	3.44	4.14	55.1
CF2.0	1.24	3.44	3.10	41.4

CoFe_2_O_4_/GQDs were synthesized in a similar manner. A mixture of Co(NO_3_)_2_·6H_2_O (4.95 g), Fe(NO_3_)_3_·9H_2_O (3.44 g), and starch (C_6_H_10_O_5_)*_n_* (6.20 g) with a *n*Co/*n*Fe/starch/*n*H_2_O molar ratio of 2:1:4.5:540 was dissolved in a flask and heated to 80–85 °C for 1 h to hydrolyse the starch. Then, the mixture was transferred to a 100 mL Teflon-line autoclave and heated at 140, 180, and 200 °C for 24 h. The brown solid was centrifuged, washed several times with distilled water, and dried at 100 °C for 24 h to obtain CoFe_2_O_4_/GQDs. The material was denoted as CF/GQDs-140, CF/GQDs-180, and CF/GQDs-200, where the numeral symbol presents the hydrolysis temperature.

For the sake of comparison, GQDs were synthesized similarly at 200 °C without cobalt and iron. The product mixture was evaporated at 100 °C for 48 h and dried to obtain GQDs.

### Photocatalysic studies

#### Kinetic study

The photochemical activity of the material was evaluated through the photochemical decomposition of MB solution under simulated sunlight conditions (160 W bulb, Osram, Germany). 0.05 g of the sample material was dispersed in 100 mL of 10 ppm MB solution in a 250 mL Erlenmeyer flask. Before illumination, the solution was placed in the dark for 1 h under stirring to achieve an adsorption–desorption equilibrium. Aliquots of 2 mL were taken at certain time intervals during irradiation and separated by centrifuging. In the supernatant, the MB concentration was determined via absorption spectroscopy at a wavelength of 664 nm. The photochemical degradation efficiency of MB was calculated according to formula 
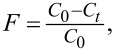
 where *C*_0_ and *C_t_* are the MB concentration at the beginning and at time *t*, respectively.

#### Effect of pH value

The CoFe_2_O_4_/GQDs composite material was placed in flasks sealed with aluminium foil and containing 20 mL of 10 ppm MB solution at pH 3–11, adjusted with 0.01 M HCl or 0.01 M NaOH. After 60 min of adsorption and photodegradation, the tubes were placed in a photocatalytic cabinet for illumination for 120 min. Then, the material was separated from the solution, and the MB concentration was determined to evaluate the MB decomposition efficiency.

#### Reusability

Reusability and stability of the CoFe_2_O_4_/GQDs were assessed in three cycles of 120 min. After each cycle, the catalyst was separated from the MB solution with a magnet and dried at 100 °C for 2 h for use in the subsequent cycle. The photocatalytic reaction in each cycle was conducted under the same conditions.

#### Scavengers

1,4-Benzoquinone (BQ, 10 mM), isopropanol (IPA), potassium iodide (KI, 10 mM), and potassium bromate (KBrO_3_, 10 mM) were used as scavengers for 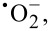 ^•^OH, h^+^, and e^−^ species in the reaction. After adding 0.05 g of CoFe_2_O_4_/GQDs material to 100 mL of a 10 ppm MB solution, the mixture was kept in the dark and stirred continuously for 60 min. Then, 10 mL of each scavenger solution was added, and the illuminator was switched on. After specified time intervals, 10 mL of the sample was taken and centrifuged to separate the material from the solution. The concentration of remaining MB in the solution was determined by using UV–vis spectroscopy.

#### Identification of intermediates

Intermediate products and mineralization level of the MB photodegradation were evaluated by using dual liquid chromatography–mass spectrometry (LC/MS-MS) after the MB solution samples were illuminated for 0, 60, 120, and 180 min. The chemical oxygen demand (COD) of MB solution samples was assessed after 0, 30, 60, 90, 120, and 150 min of light exposure.

## Result and Discussion

### Materials characterisation

**Figure 1:**
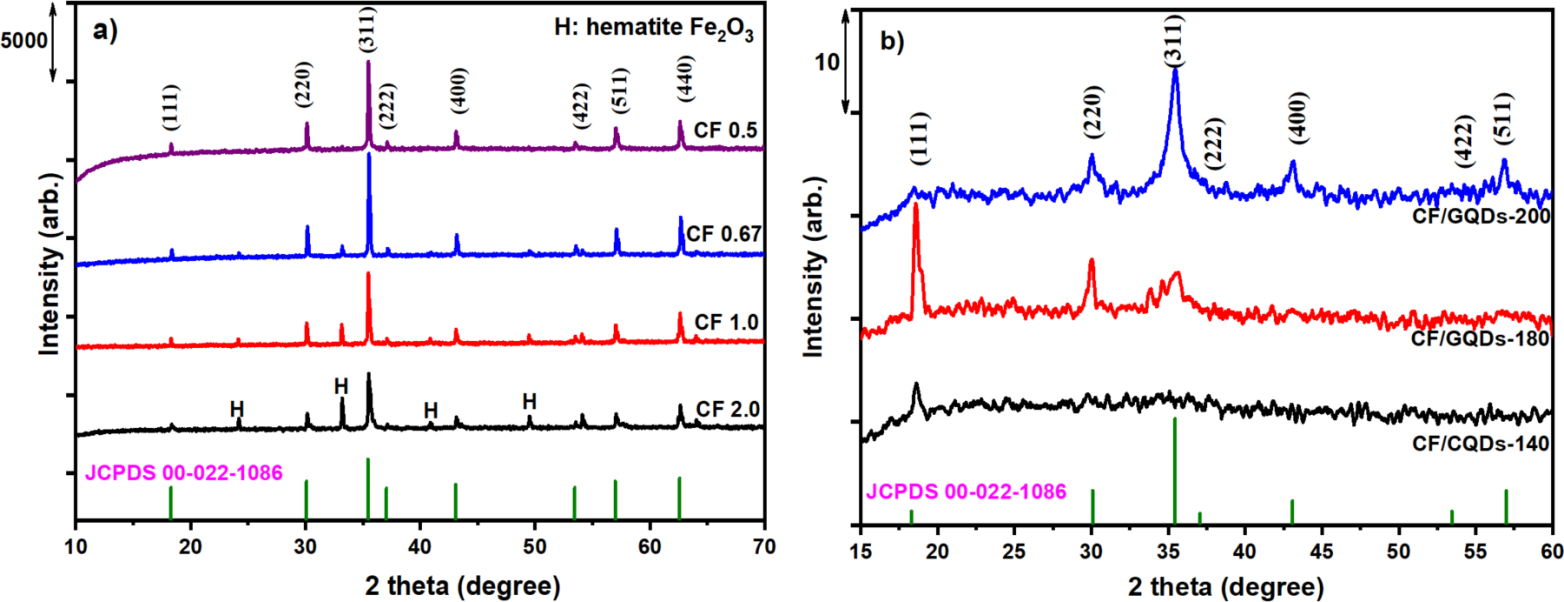
XRD patterns of (a) CF and (b) CF/GQDs.

The XRD patterns of cobalt ferrite with decreasing Fe/Co molar ratio from 2 to 0.5 are presented in Figure 1a. The XRD patterns of CF confirm that cobalt ferrite has a spinel structure, and all main peaks correspond to the standard pattern of bulk CoFe_2_O_4_ (JCPDS 00-022-1086). It is notable that the XRD pattern of cobalt ferrite with the initial Fe/Co ratio of 2 (stoichiometric ratio) exhibits the diffraction of iron oxides at 2θ of 24.2°, 33.2°, 40.8°, and 49.6° (JCPDS no. 33-0664). This means that a part of cobalt ions goes to the liquid phase during synthesis. Therefore, it is necessary to ensure a higher amount of cobalt than its theoretical ratio to achieve stoichiometric cobalt ferrite. Cobalt ferrite with the initial Fe/Co molar ratio of 0.5 has also spinel structure, and no additional and intermediate phase are observed, indicating that a single-phase spinel was obtained. Therefore, the initial Fe/Co molar ratio of 0.5 was used to prepare CF/GQDs. Figure 1b presents XRD patterns of CF/GQDs prepared at different temperatures. No diffraction peaks of CF/GQDs prepared at 140 °C are observed; the characteristic peaks of cobalt ferrite spinel appear for the samples prepared at 180 and 200 °C. No signals for GQDs can be observed because of the low crystallinity, high dispersion, and small amount of GQDs in CF/GQDs.

The peak of the (222) plane is related to the octahedral sites, while the peaks at the (220) and (422) planes are attributed to the tetrahedral sites [13]. The ratio of the peak intensities between the (220) and the (222) planes as well as between the (422) and the (222) planes are used to estimate the distribution of cobalt ions in the tetrahedral sites or octahedral sites. The values of the intensity ratios (*I*_220_/*I*_222_) and (*I*_422_/*I*_222_) for CF and CF-GQDs-200 are 1.25 and 1.05, and 1.09 and 1.00, respectively. The ratios in CF are higher than those in CF-GQDs, indicating that the number of cobalt ions at tetrahedral sites in CF is higher than that in CF/GQDs-200.

The crystallite size of the as-prepared CoFe_2_O_4_ ferrite particles was calculated from the (311) peak of the XRD pattern by using the Scherrer equation [14], 
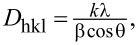
 where *D*_hkl_ is the crystallite size; *k* is the shape factor (0.89); θ is the diffraction angle; β is the full width at half maximum of the (311) peak, and λ is the X-ray wavelength (1.54 Å). The average crystallite sizes of the as-synthesized CoFe_2_O_4_ and CF/GQDs-200 are, respectively, about 17.2 and 9.2 nm. Since cobalt ferrite possesses an inverse spinel structure (space group 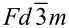) [13], the ferrite lattice parameter of CF and CF/GQDs-200 is 8.394 Å, agreeing well with the bulk value of 8.377 Å [15].

**Figure 2:**
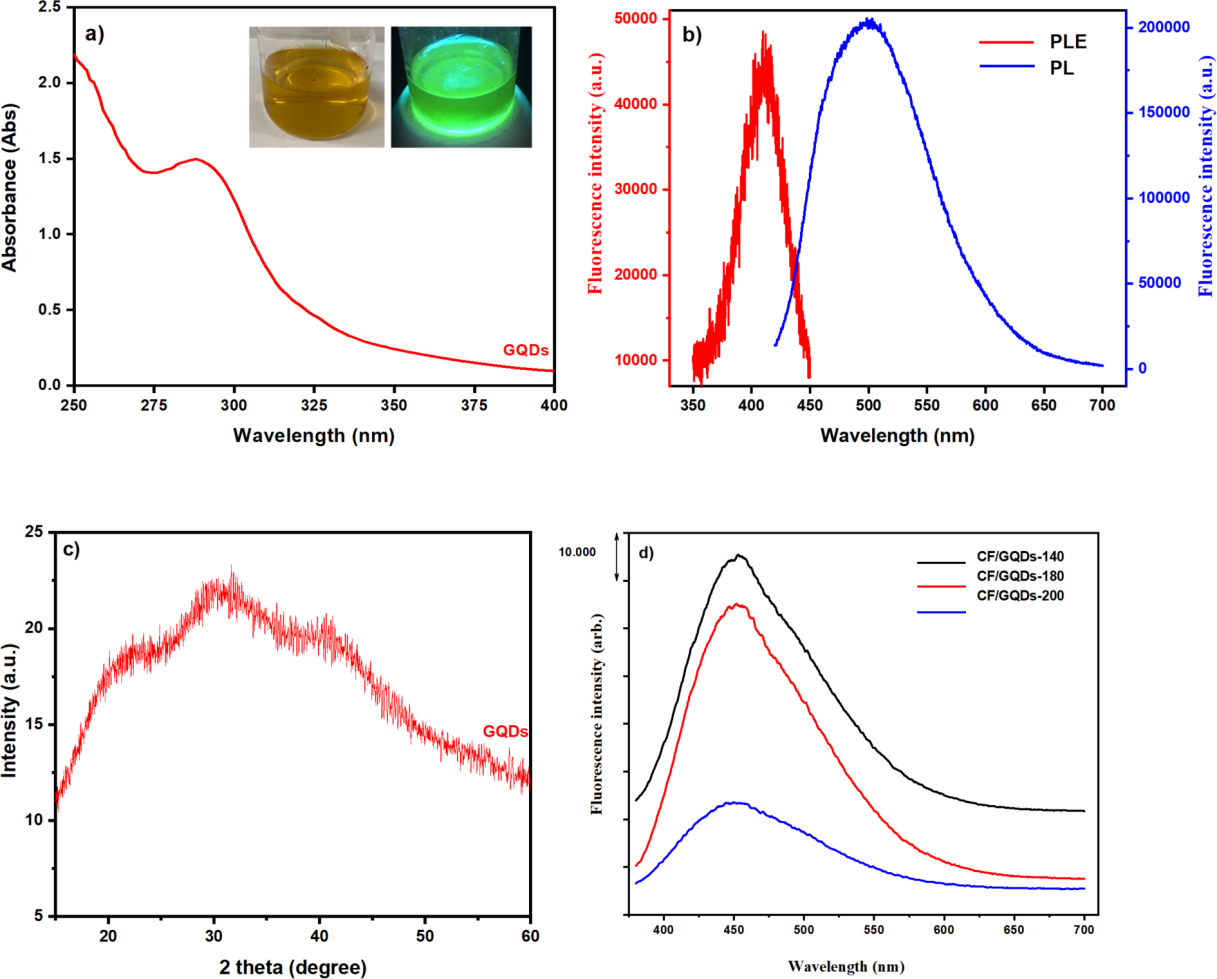
(a) UV–vis absorption spectra of the GQD solution; inset: GQD solution under white light (left) and ultraviolet light (254 nm, right); (b) photoluminescence excitation (PL) and photoluminescence emission spectra (PLE) of the GQDs; (c) XRD pattern of GQDs; (d) PL spectra of supernatants from prepared CF/GQD suspensions.

The suspension of CF/GQDs-200 after thermal hydrolysis was filtered by centrifuging and divided into two parts, that is, solution and solid. The optical properties of the solution are shown in Figure 2a. The inset in Figure 2a presents the 100 mL beaker containing the GQD solution. The sample on the left was exposed to white light, and it has a yellow-brown colour, characteristic of GQDs prepared from the natural polymer starch [16]. The solution on the right was exposed to 254 nm UV light, and the colour changed to green. The typical absorption peak at 287 nm in the UV–vis spectrum of the aqueous GQD solution (Figure 2a) can be attributed to the n–π* transition of graphitic sp^2^ domains, which is the characteristic band of a polyaromatic structure [16–17]. Figure 2b presents the maximum emission at a wavelength of 498 nm (blue line) with an excitation wavelength of 410 nm (red line). The exact PL behaviour of GQDs is still unclear. It is possible that electron–hole recombination, quantum effects, and surface defects in the functional groups of the GQDs are involved [18]. The XRD pattern of GQDs prepared from starch without iron and cobalt salts is presented in Figure 2c. The diffraction peaks of the GQDs are centred at 2θ of 23°, 30.9°, and 39.6°, corresponding to d-spacing values of 0.52, 0.45, and 0.21 nm, respectively. A larger d-spacing value manifests that GQDs contain oxygen functional groups. This could be assigned to the size of GQDs. The broad nature of the diffraction peaks is attributed to GQDs with nanoscale size composed of few-layer graphene. These results confirm that the supernatant contains GQDs. Similarly, the supernatants from the CF/GQDs suspensions synthesized at 140 and 180 °C (Figure 2c) also contain graphene quantum dots (Figure 2c).

**Figure 3:**
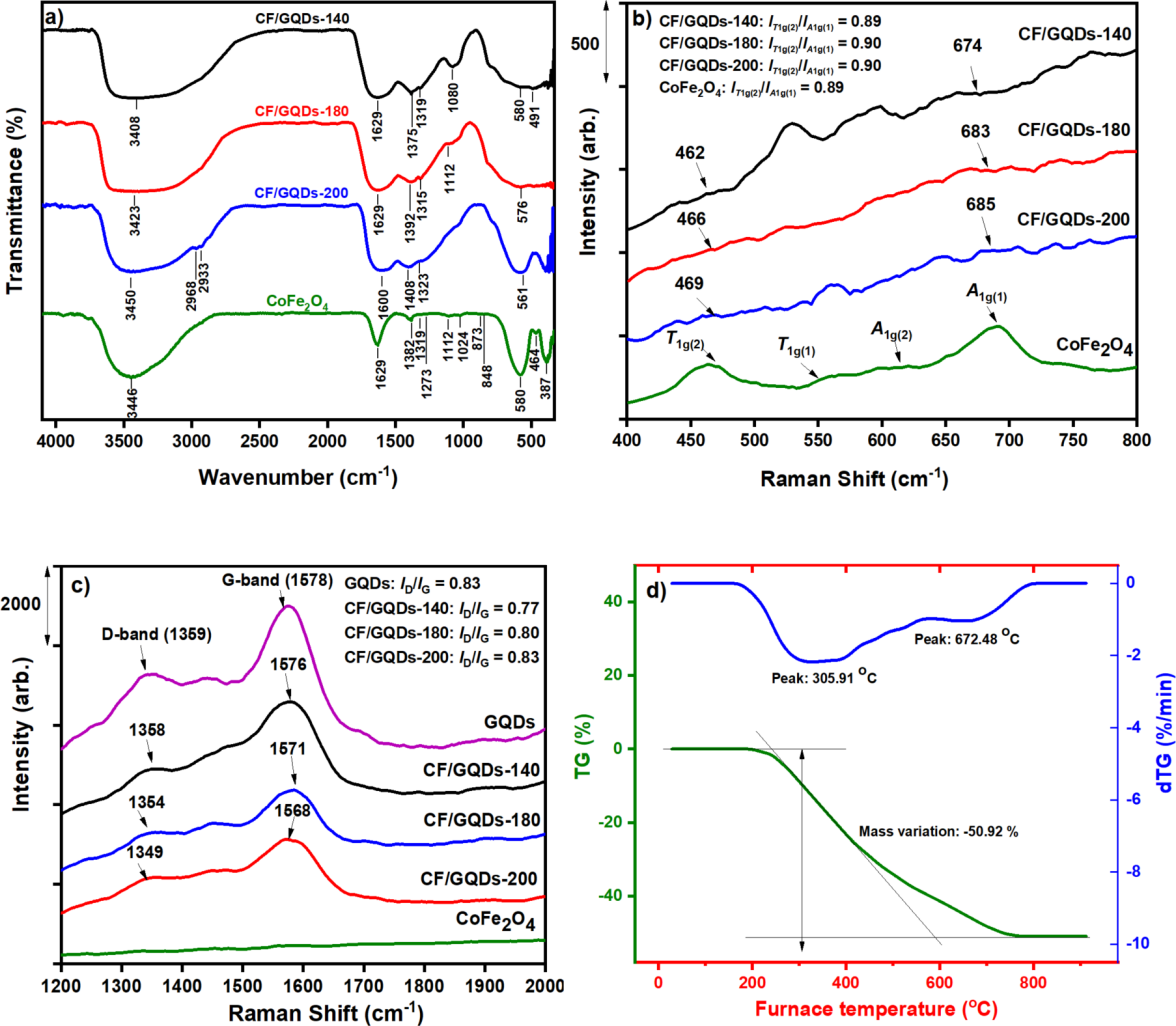
(a) FTIR spectra of CF/GQDs and CoFe_2_O_4_; (b, c) Raman spectra; (d) TG/dTG curves of CF/GQDs-200.

Figure 3 presents some characterisations of the solid part extracted from the suspension of CF/GQDs-200. The FTIR spectra of CF and CF/GQDs-200 are shown in Figure 3a. CF has two broad bands at 387 and 580 cm^−1^, attributed to the characteristic stretching vibrations of the iron and cobalt bonds at the tetrahedral and octahedral sites, respectively [19]. For CF/GQDs, the broad bands from 750–400 cm^−1^ possibly involve the vibrations of metal and oxygen in cobalt ferrite. The bands at 3408–3450 cm^−1^ are attributed to stretching vibrations of O–H groups, while the bands at 1622, 1375, 1233, and 1080 cm^−1^ are assigned to C=C vibrations of aromatic carbons, O–H bending in carboxylic and carbonyl groups, C=O vibrations in epoxy groups, and stretching vibrations of C–O in alkoxy groups, respectively [20–21]. These functional groups prove the existence of GQDs in CF/GQDs. The Raman spectra of CF and CF/GQDs are presented in Figure 3b,c. CF exhibits four peaks at 469, 550, 614, and 690 cm^−1^, corresponding to the *T*_1g(2)_, T_1g(1)_, *A*_1g(2)_, and *A*_1g(1)_ Raman modes, respectively [22]. For CF/GQDs, these modes are broad and weak, but these results also confirm the existence of CF in GQDs.

In contrast to the spectra of pure CF, there are obvious vibrations at 1200–2000 cm^−1^ with two characteristic peaks of graphitic carbon (G band) and disordered carbon (D band), appearing at 1590 and 1298 cm^−1^, respectively. The *I*_D_/*I*_G_ ratio is 1.03, which is higher than that of graphene oxide (GO), which is 0.93. The strong band in the Raman spectra at 690 cm^−1^ is attributed to the *A*_1g(1)_ symmetry (tetrahedral sites) [22], and the band at 469 cm^−1^ is assigned to the *T*_1g(2)_ symmetry at the octahedral sites [19]. The ratio of intensities of these two peaks at 469 and 690 cm^−1^ also reveals the distribution of Co^2+^ between the tetrahedral and octahedral sites [13]. This peak intensity ratio for CF is higher than that for CF/GQDs-200. These results from Raman spectra are consistent with XRD analysis, indicating that Co^2+^ in CF has a tendency to settle at tetrahedral sites, while Co^2+^ in CF/GQDs-200 does not.

The TG-dTG curves of CF/GQDs-200 is presented in Figure 3d. Weight losses are observed between 200 and 800 °C, and the material remains stable at higher temperatures. The significant loss from 200 to 700 °C of around 50.92% is attributed to GQDs. These results show that the equation illustrating the synthesis of CF/GQDs can be written as follows:




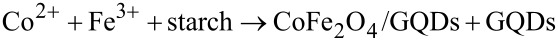




**Figure 4:**
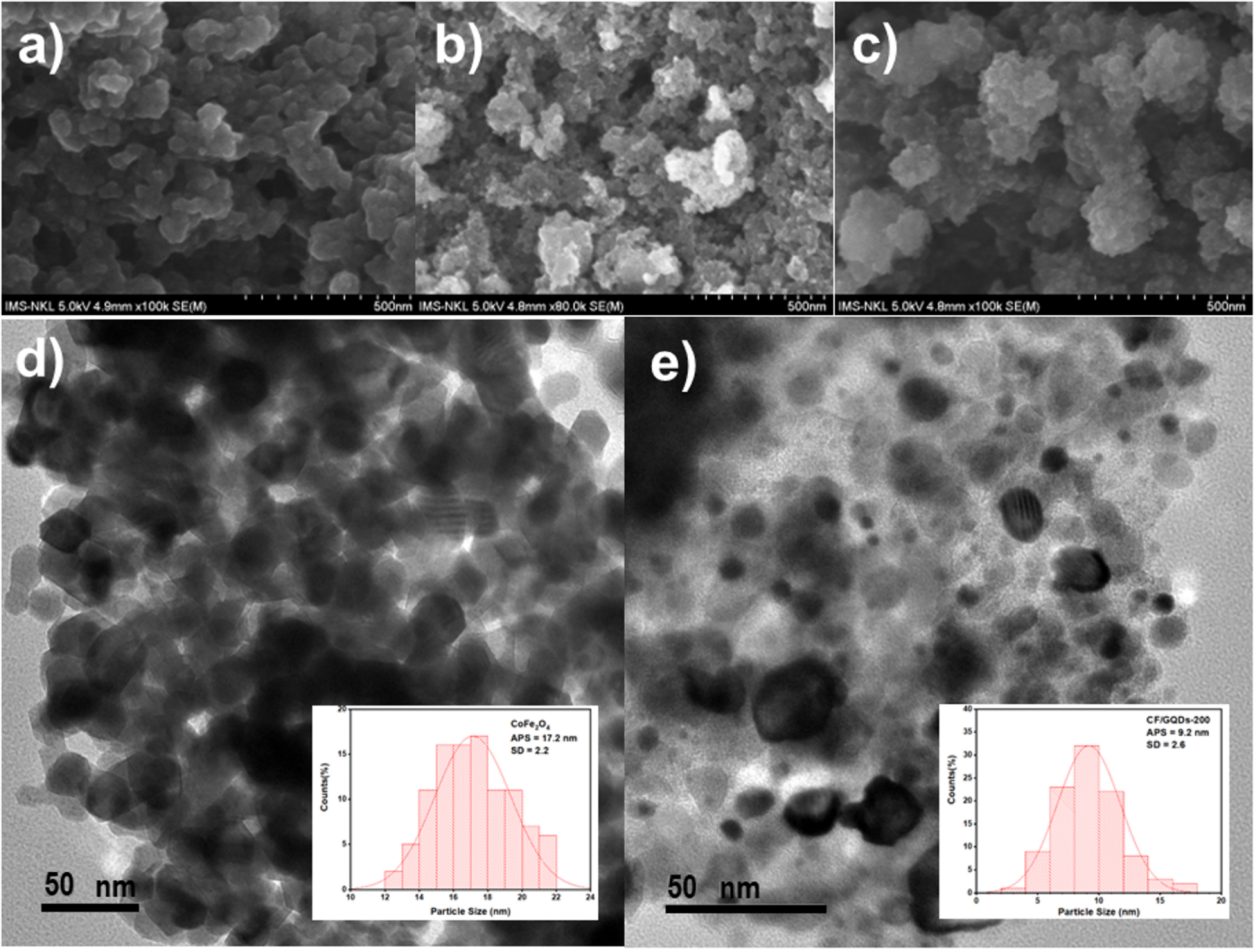
SEM images of (a) CF/GQD-140, (b) CF/GQD-180, and (c) CF/GQD-200; TEM observations and corresponding particle size distribution of (d) CF and (e) CF/GQDs-200.

SEM images of CF/GQDs synthesized at hydrothermal temperatures of 140, 180 and 200 °C are shown in Figure 4a–c. The morphology reveals that CF/GQDs consist of heavily agglomerated particles, several hundreds of nanometers in diameter, because of their magnetic nature. Figure 4d and Figure 4e present the TEM images of CF and CF/GQDs-200, respectively, and the corresponding particle size distribution. CF has very fine particles of around 15–20 nm. The intimate interfacial contact between GQDs sheets and the CF nanoparticle is further depicted in the TEM image (Figure 4e). In this image, the deposited nanoparticles of around 9.2 nm are obvious on the GQDs. Such a good interfacial contact between CF and GQDs is favourable for the separation of the photogenerated charge carriers in the CF/GQDs.

**Figure 5:**
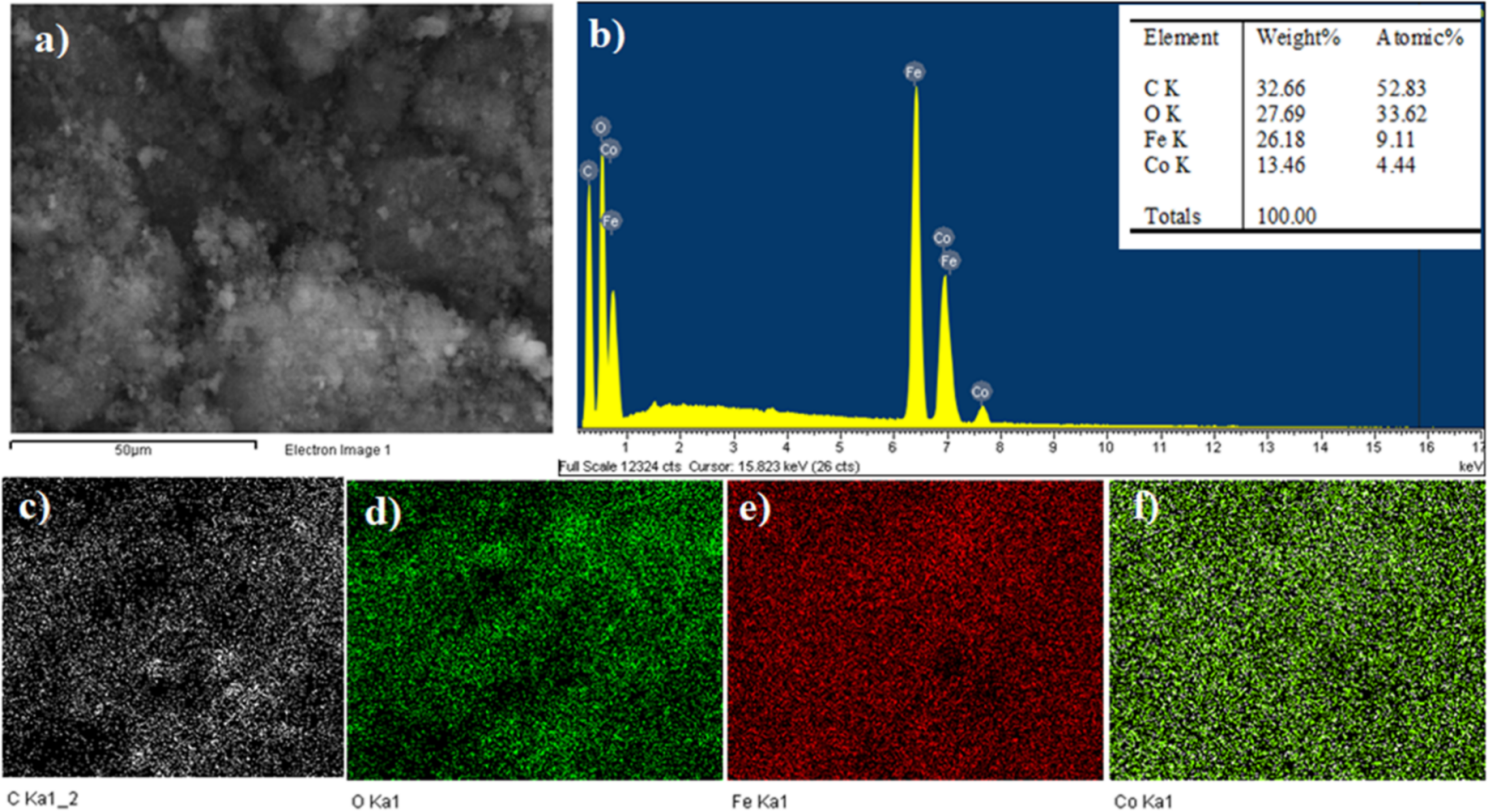
EDX-mapping of CF/GQDs-200. (a) Electron microscopy image, (b) EDX spectrum, (c) carbon mapping, (d) oxygen mapping, (e) iron mapping, and (f) cobalt mapping.

The elemental distribution of CF/GQDs was studied by EDX mapping. The elements Fe, Co, O, and C are present, as expected. Figure 5 shows that the elements, such as iron and cobalt, are evenly distributed in the GQDs matrix. No other elements were found, indicating no impurities in CF/GQDs. The Fe/Co molar ratio of 2.05 in CF/GQDs is very close to 2, the Fe/Co initial ratio.

**Figure 6:**
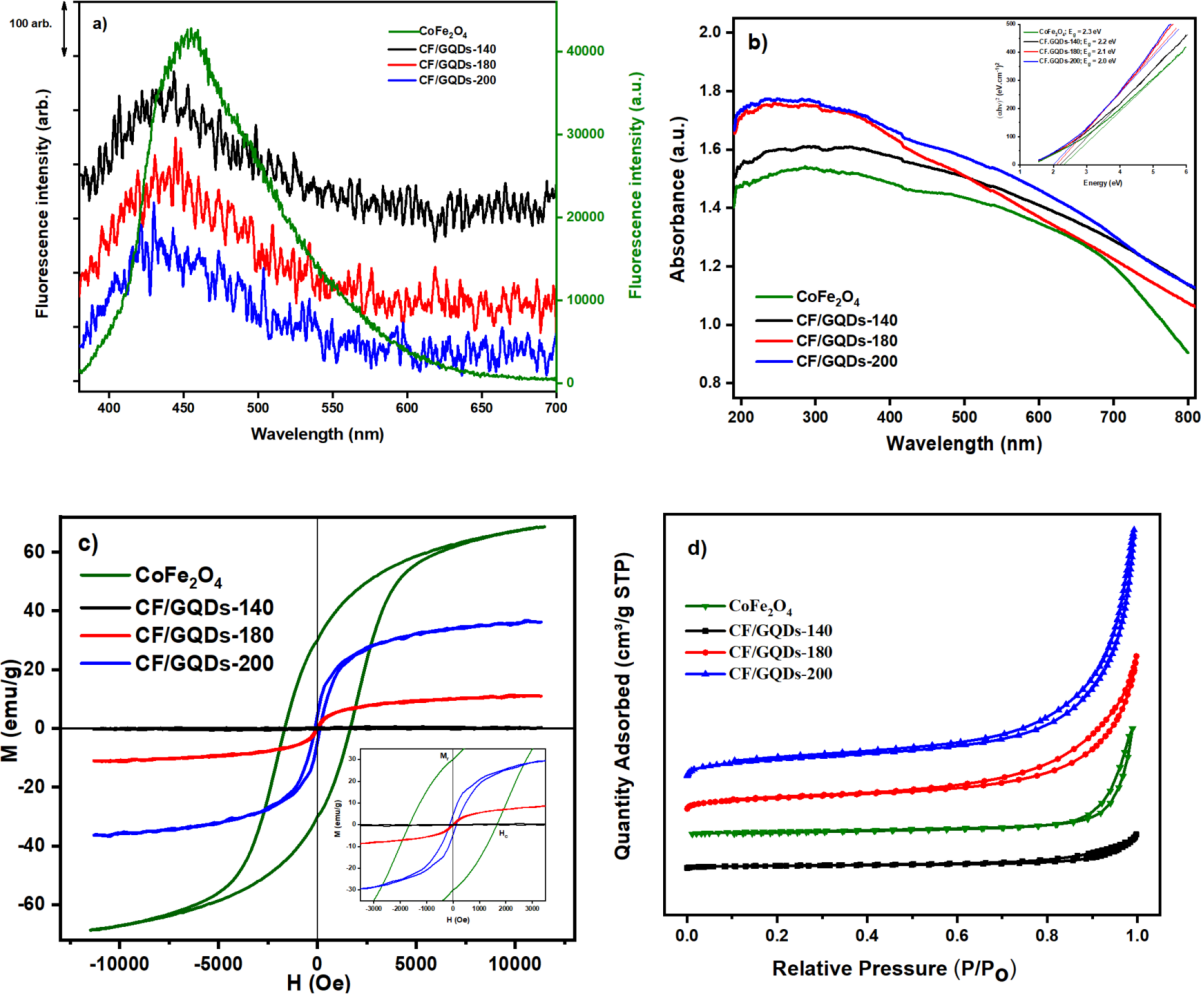
(a) PL spectra, (b) UV–vis diffuse reflectance spectra with Tauc’s plots in the inset, (c) magnetization curves, and (d) nitrogen adsorption/desorption isotherms of CF/GQDs prepared at different temperatures.

As depicted in Figure 6a, the CF/GQDs nanoparticles exhibit a significantly lower PL emission intensity than CF alone because of the higher separation efficiency of charge carriers of the CF/GQDs sample. The UV–vis DRS spectra of CF and CF/GQDs (Figure 6b) show that the absorption intensity becomes stronger after grafting the GQDs on CF, indicating that the resulting composite can enhance the absorption capacity [11]. This observation is an agreement with previous works [11]. In addition, a slight shift of the PL emission peak is also observed because of the blue shift in the UV–vis spectrum, which is consistent with their bigger bandgap energy.

The magnetic characteristics of the CF/GQDs nanocomposite were identified by using VSM. The magnetic parameters, including the saturation magnetization (*M*_s_), coercivity (*H*_c_), and remanence (*M*_r_), are presented in Table 2. As can be seen in Figure 6c, the hysteresis characteristics of CoFe_2_O_4_ show hard ferrites with high coercive field and magnetization, while the hysteresis loops of CF/GQDs are reversible, indicating that CF/GQDs exhibit superparamagnetic characteristics. The magnetic saturation values of the CF/GQDs-140, -180 and -200 samples are 0, 11.1, and 36.3 emu/g, respectively, at room temperature. They are much smaller than the bulk value (65.8 emu/g). The decrease in the saturation magnetization of the CF/GQDs is attributed to the presence of the nonmagnetic GQDs. The particles’ high magnetic permeability shows that they could be separated with an external magnetic field. The *M*_r_ values of 0.6 emu/g for CF/GQDs-180 and 4.6 emu/g for CF/GQDs-200 are taken from the inset in Figure 6c. The obtained *H*_c_ value (146.6 Oe) for our sample is much lower than that in the literature [23]. Generally, the CoFe_2_O_4_ ferrite particles may have a multidomain structure. The formation of multiple domains and the easy movement of the domain walls can result in a decrease in coercivity. The reduced particle size leads to reduced coercivity, which could be expressed as a transformation from a ferrimagnetic to a superparamagnetic state [15].

Figure 6d presents the nitrogen adsorption/desorption isotherms of CoFe_2_O_4_ and CF/GQDs. All isotherm curves belong to type V according to IUPAC. At a high relative pressure (above 0.8), the hysteresis sloop indicates that the materials have micro-meso structures. The surface area of CoFe_2_O_4_ is small because of heavy agglomeration (21.0 m^2^·g^−1^). However, CF/GQDs have a significantly larger surface area compared with that of CoFe_2_O_4_ (11.3 m^2^·g^−1^ for CF/GQDs-140, 59.3 m^2^·g^−1^ for CF/GQDs-180, and 85.2 m^2^·g^−1^ for CF/GQDs-200). This is possibly due to stacked GQDs in CoFe_2_O_4_ crystals, making the structure of CF/GQDs more porous. The surface area increases with increasing hydrolysis temperature because the higher the temperature, the more the CoFe_2_O_4_ crystals form, making it less agglomerated. The CF/GQDs-200 sample with the largest surface area was selected for further studies.

**Table 2:** Physical properties of CF/GQDs.

Sample	*S*_BET_ (m^2^·g^−1^)	Porous volume (cm^3^·g^−1^)	Crystallite size (nm)	*M*_s_ (emu·g^−1^)	*M*_r_ (emu·g^−1^)	*H*_c_ (Oe)

CoFe_2_O_4_	21.0	0.17	32.5	65.8	30.0	1654.9
CF/GQDs-140	11.3	0.05	—	—	—	—
CF/GQDs-180	59.3	0.24	—	11.1	0.6	38.4
CF/GQDs-200	85.2	0.41	9.2	36.3	4.6	146.6

### Photocatalytic studies

**Figure 7:**
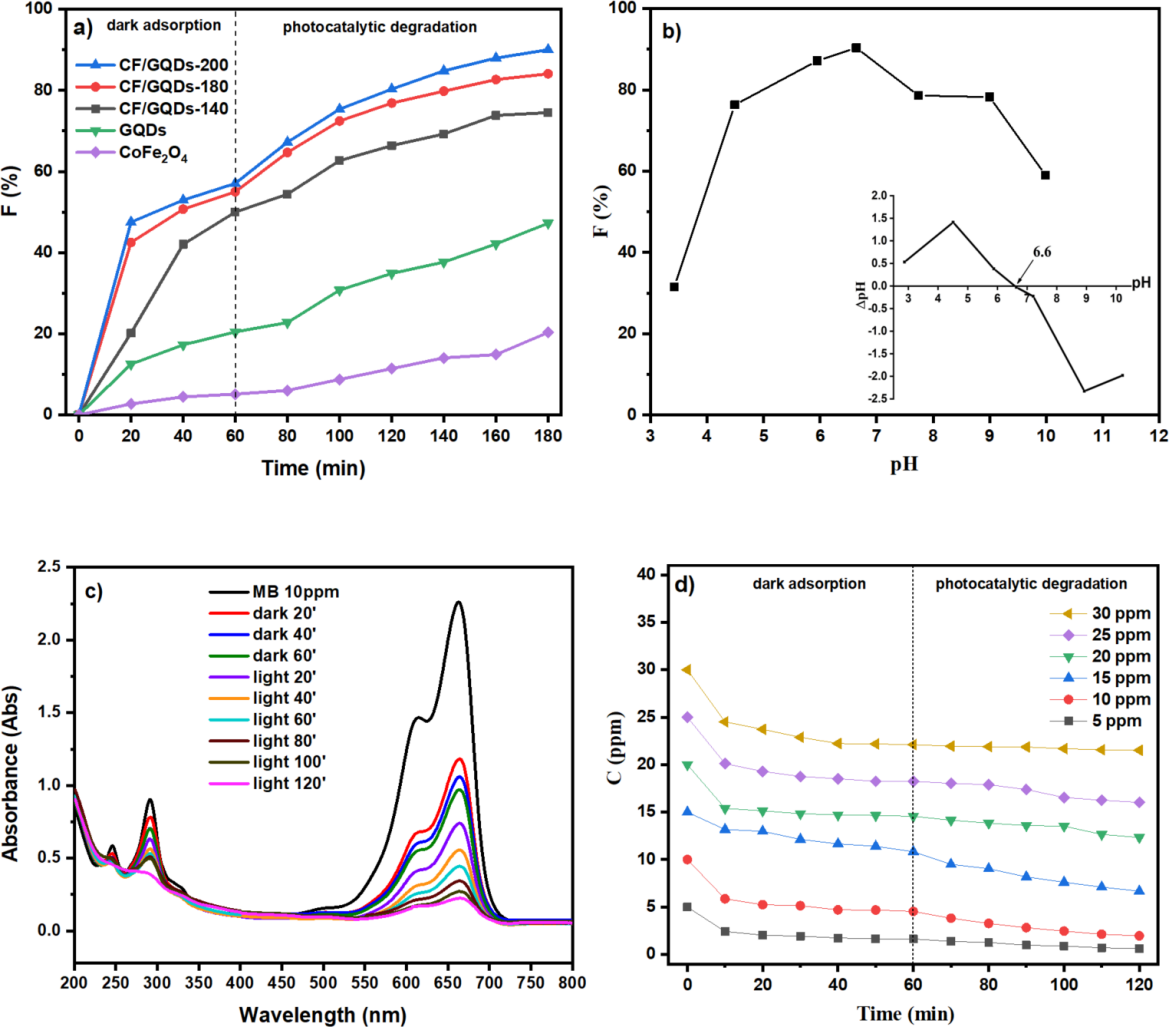
(a) MB decolourisation over different catalysts (*V* = 100 mL, *C*_0(MB)_ = 10 ppm, *m* = 0.05 g, time of darkness adsorption: 60 min; time of light irradiation: 120 min). (b) Effect of pH value on the MB decolourisation over CF/GQDs-200; the inset presents the point of zero charge (*V* = 20 mL, *C*_0(MB)_ = 10 ppm, *m* = 0.02 g, time of light irradiation: 120 min). (c) UV–vis spectra of MB and the treated solution (*V* = 100 mL, *C*_0(MB)_ = 10 ppm, *m* = 0.05 g, time of darkness adsorption: 60 min, time of light irradiation: 120 min). (d) Decolourisation kinetics (*V* = 100 mL, *C*_0(MB)_ = 5–30 ppm, *m* = 0.05 g, time of darkness adsorption: 60 min, time of light irradiation: 60 min).

Figure 7a presents the decolourisation kinetics over different CF/GQDs samples as catalysts. The decolourisation occurs in two steps. First, the catalyst was mixed with the MB solution in the dark for 60 min to ensure the adsorption/desorption equilibrium; second, the lamp was turned on to irradiate the MB solution under mechanical stirring. It is found that the rate constant increases in the following order: CF/GQDs-200 (*k* = 0.0123 min^−1^) > CF/GQDs-180 (*k* = 0.0087 min^−1^) > CF/GQDs-140 (*k* = 0.0059 min^−1^) > GQDs (*k* = 0.0034 min^−1^) > CF (*k* = 0.0014 min^−1^).

The CoFe_2_O_4_ particles agglomerate heavily because of their magnetic nature, as expected, and they exhibit slight MB adsorption and decolourisation. The combination of GQDs and CoFe_2_O_4_ improves the decolourisation capacity significantly. Stacking CoFe_2_O_4_ crystals onto GQDs sheets not only prevents agglomeration but also creates heterojunction contacts or intermediate steps in the electronic structure. These heterojunctions limit the recombination of photo-induced electron–hole pairs and, thus, enhance catalytic decolourisation. Therefore, the CF/GQDs-200 sample was selected for further studies.

The point of zero charge (pzc) of CF/GQDs-200 obtained by the pH drift method is 6.6 (see the inset of Figure 7b). Therefore, the surface of CF/GQDs-200 is negatively charged at pH > 6.6 and positively charged when pH < 6.6. Methylene blue possesses three basic sites, whose p*K*_a_ values are 2.6 (p*K*_a1_), 11.2 (p*K*_a2_), and 11.2 (p*K*_a3_) [24]. Based on the average p*K*_a_ value of MB, the point of zero charge for MB is calculated as 8.33 [25]. The MB dye can exist in several chemical forms, namely MBH_3_^2+^ (the tri-protonated form), MBH_2_^+^ (diprotonated form), MBH (monoprotonated form), and MB^−^ (deprotonated form) [25].

With increasing pH, the decolourisation efficiency increases, reaches its maximum at pH ca. 7, and decreases afterwards (Figure 7b). This could possibly be due to the fact that at low pH, the increase in pH makes the surface of CF/GQDs and MB become less positive, and the attractive interaction increases. In contrast, at high pH, the increasing pH makes the surface of CF/GQDs and MB become more negative; as a result, the repulsion force of these species comes into play, reducing adsorption.

UV–vis spectroscopy was used to study MB decolourisation (Figure 7c). The UV–vis spectra of MB present a strong adsorption band at 664 nm, corresponding to functional groups (n–π* electron transition), and two adsorption bands at 246 and 292 nm corresponding to aromatic rings (π–π* electron transition). The colour intensity of the MB solution decreases only slightly in the absence of catalysts (0 to 10 h), indicating that MB is stable and exhibits weak self-degradation under incident radiation. However, when CF/GQDs-200 was added, the peak magnitude for MB at 246, 292, and 664 nm decreased remarkably. The colour disappearance and the cleavage of aromatic rings were completed after 120 min of irradiation (Figure 7c). The decolourisation kinetics of MB over CF/GQDs-200 is shown in Figure 7d. The Langmuir–Hinshelwood first-order kinetic model is widely employed to assess the kinetic data of photocatalytic degradation. This model is as follows: 
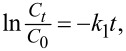
 where *C_t_* and *C*_0_ are the MB concentration at the beginning and at the time *t*, respectively; *k*_1_ is the first-order kinetic rate constant. The slope of the linear plot of 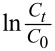 vs *t* provides the value of *k*_1_. The values of *k*_1_, along with others from the literature, are presented in Table 3. However, because the reactions were conducted under different conditions, the comparison is arbitrary.

**Table 3:** A comparison of the rate constant of the present work with other values from literature.

Materials	Mass of catalyst (mg)/mass of dye (mg)	Light source	*k* (min^–1^)	Ref

Co_0.5_Zn_0.5_Fe_2_O_4_	0.5/1	visible light	0.064	[26]
CoFe_2_O_4_	1/1	visible light	0.01	[27]
ZnFe_2_O_4_	200/1	UV–vis light	0.032	[28]
Co_0.5_Mg_0.5_Fe_2_O_4_	200/1	UV–vis light	0.072	[28]
Zn_0.25_Co_0.375_Mg_0.375_Fe_2_O_4_	200/1	UV–vis light	0.108	[28]
ZnFe_2_O_4_/Fe_2_O_3_	100/1	75 W mercury lamp	0.019	[29]
MnFe_2_O_4_/rGO	30/1	UV–vis light	0.0586	[30]
γ-Fe_2_O_3_/Fe_3_O_4_/ SiO_2_	2/1	UV light	0.01836	[31]
g-C_3_N_4_/Ca_2_Fe_2_O_5_	156/1	natural sunlight	0.058	[32]
Co_0.1_Mg_0.9_Fe_2_O_4_	20/1	halogen lamp	0.005	[33]
CF/GQDs	50/1	160 W Osram bulb	0.0123	this work

To clarify the mechanism of MB photocatalytic degradation, 10 mM isopropyl (IPA), potassium iodide (KI), potassium bromate (KBrO_3_), and *p*-benzoquinone (BQ) were used as scavengers to capture hydroxyl radicals (^•^OH), photo-induced holes (h^+^), photo-induced electrons (e*^−^*), and superoxide anions 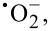 respectively. As can be seen, IPA exhibits a strong interaction with the hydroxyl radical through a simple electron transfer process [34].




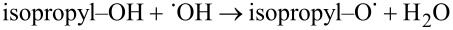




**Figure 8:**
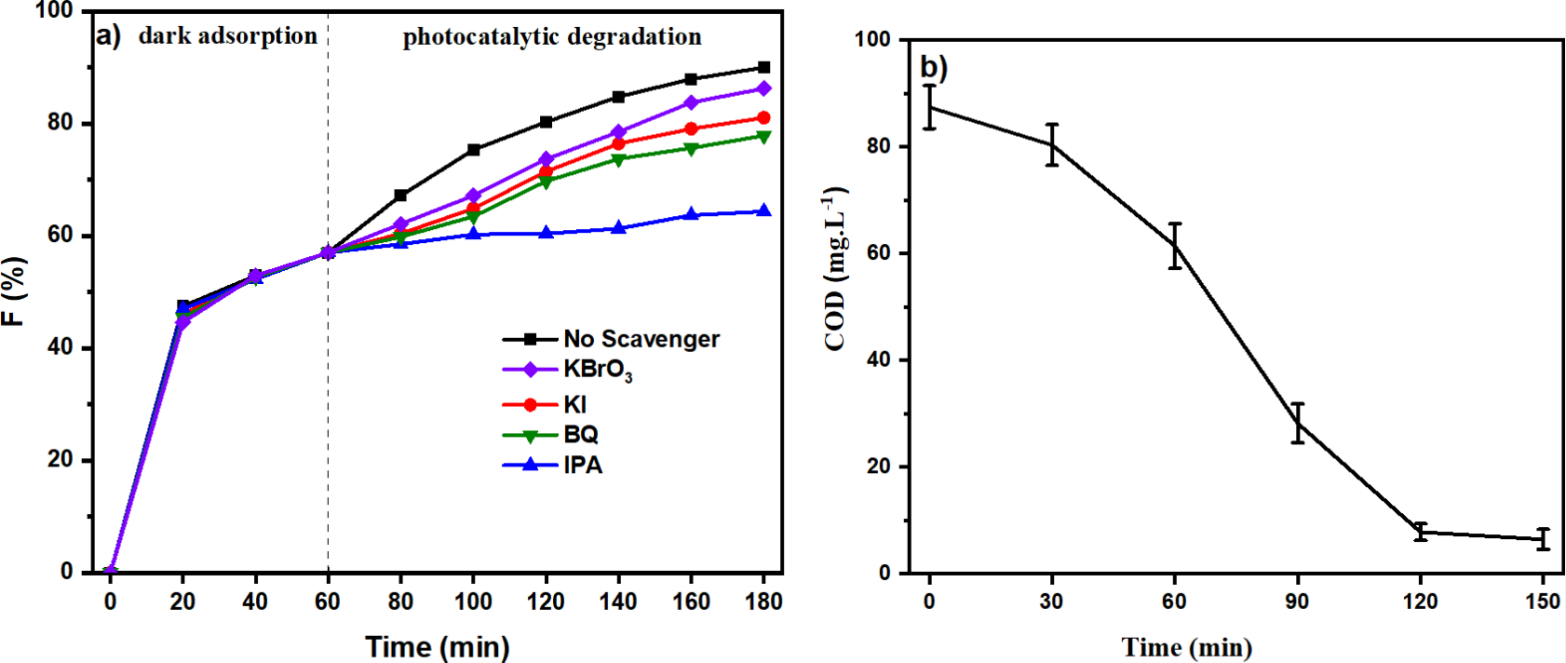
(a) Effects of addition of KI, IPA, KBrO_3_, and BQ on the visible-light-driven photocatalytic degradation of MB with CF@GQDs; (b) dependence of the chemical oxygen demand on the reaction time.

If the ^•^OH radicals play a crucial role in the MB degradation, the reaction rate is expected to decrease significantly. As depicted in Figure 8a, adding an excess amount of 10 mM IPA to the reaction mixture significantly suppresses the MB degradation (by ca. 28.5%). The rate constant of MB degradation decreases to 0.0015 min^−1^ from 0.0123 min^−1^. This result indicates that the ^•^OH degradation pathway plays a critical role in the MB photocatalytic degradation.

Iodide ions (I^−^) are strong scavengers that react with valence band holes (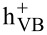) [35].




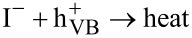




Bromate ions (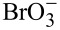) capture photoinduced electrons (e_CB_), which leads to a reduction to form bromide ions [36].




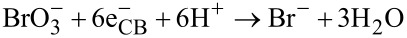




Figure 8a presents the effect of adding an excess of 10 mM KI or 10 mM KBrO_3_ on the MB degradation. The rate constant decreases to 0.0059 min^−1^ for KI and 0.0074 min^−1^ for KBrO_3_, and the decolourisation efficiency decreases by 9.9% for KI and by 4.2% for KBrO_3_ compared with the absence of scavenger. This means that the MB photocatalytic degradation is moderately affected by KI or KBrO_3_, which indicates the role of h^+^ and 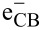 in the MB photocatalytic degradation.

*p*-Benzochinone reacts as a scavenger with superoxide anions through a simple electron transfer process [37].




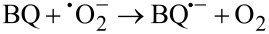




The photocatalytic degradation of MB is mildly affected by BQ (decolourisation efficiency drops by 13.5%, indicating the influence of 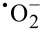 during the photocatalytic degradation of MB [37].

**Scheme 1:**
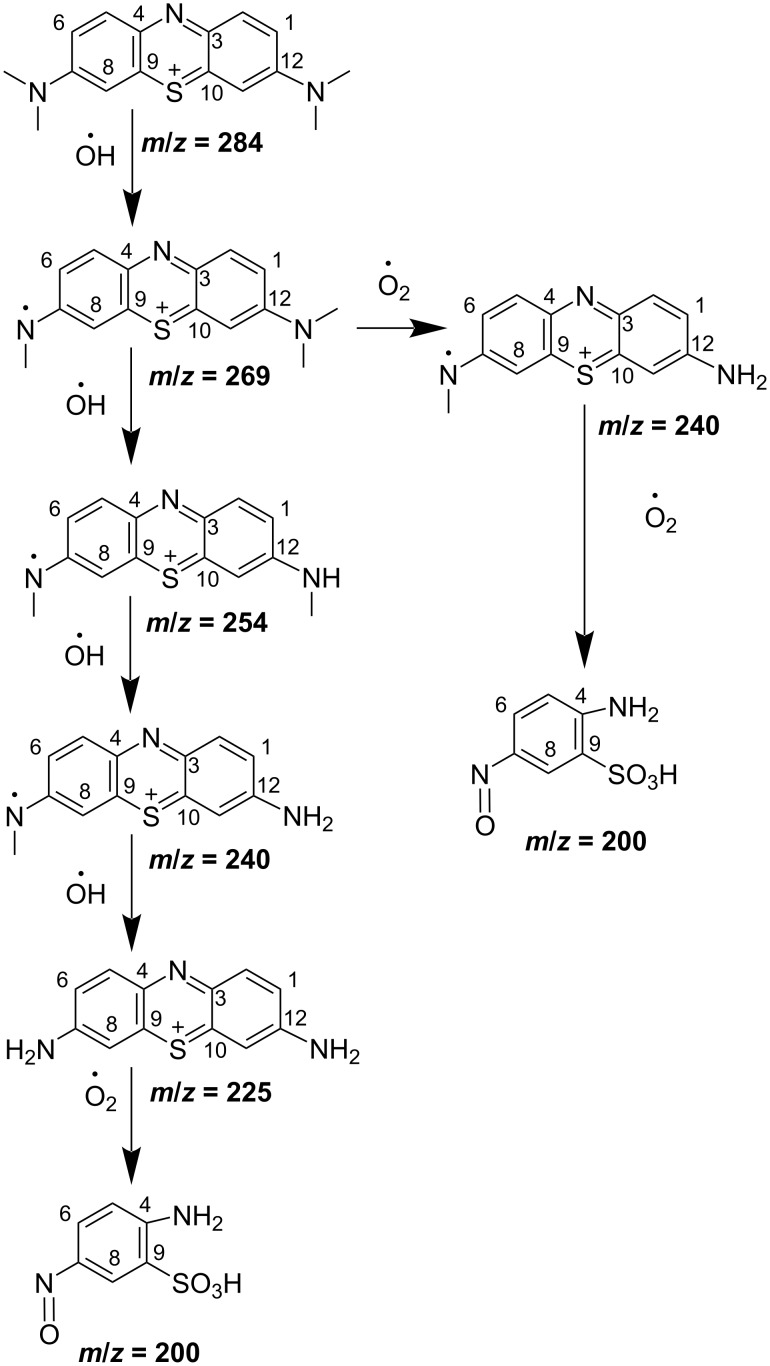
Proposed intermediates in MB degradation monitored by liquid chromatography coupled with tandem mass spectrometry recorded at 180 min (tested concentration: 10 ppm).

The mineralisation of MB was estimated with COD measurements (Figure 8b). The value of COD decreases with increasing reaction time. The initial COD value is 87.4 mg·L^−1^ and decreases to 6.5 mg·L^−1^ after 150 min of reaction, indicating that the mineralisation was almost complete. Furthermore, LC-MS was used to explore the MB degradation pathway. Detailed data is provided in Supporting Information File 1. The mass spectra of the oxidized MB solutions at different residue times show the various intermediates due to demethylation and hydroxylation processes [38]. Low-bond-energy intermediates and ring-breaking products were obtained in the MB degradation (acetates, oxalates, and sulfates, Scheme 1). Finally, MB could be mineralized to CO_2_, H_2_O, 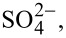 and 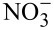 [38].

**Figure 9:**
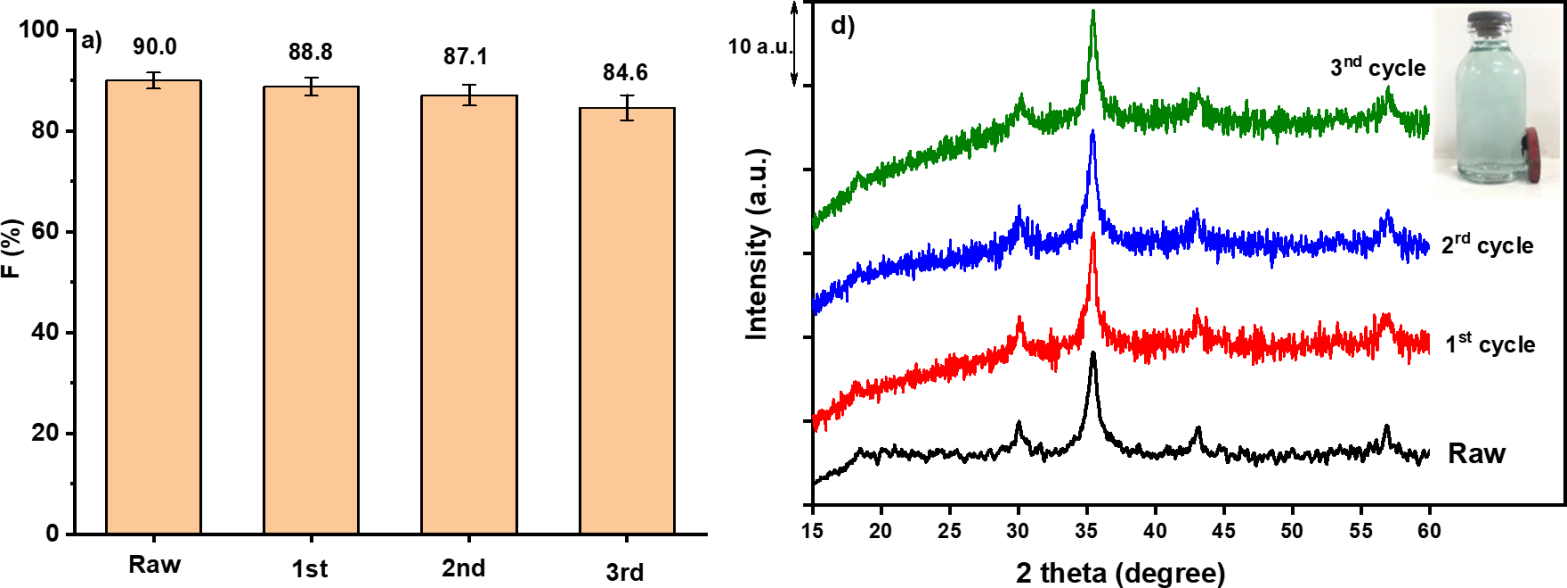
(a) Cyclic photocatalytic degradation experiments of MB with CF/GQDs-200 photocatalyst; (b) XRD pattern of CF/GQDs and reused CF/GQDs.

In order to check the stability of the materials, the prepared CF/GQDs-200 catalysts were reused in three cycles (Figure 9b). After each run, the catalyst was separated with an external magnetic field. It was found that the present photocatalyst exhibits no significant loss of activity over three cycles. The CF/GQDs-200 photocatalyst used in the cycling tests was characterised by using XRD before and after the cycling experiments. The result indicates that the as-obtained CF/GQDs-200 does not suffer from photocorrosion and exhibits excellent reusability for the degradation process.

**Scheme 2:**
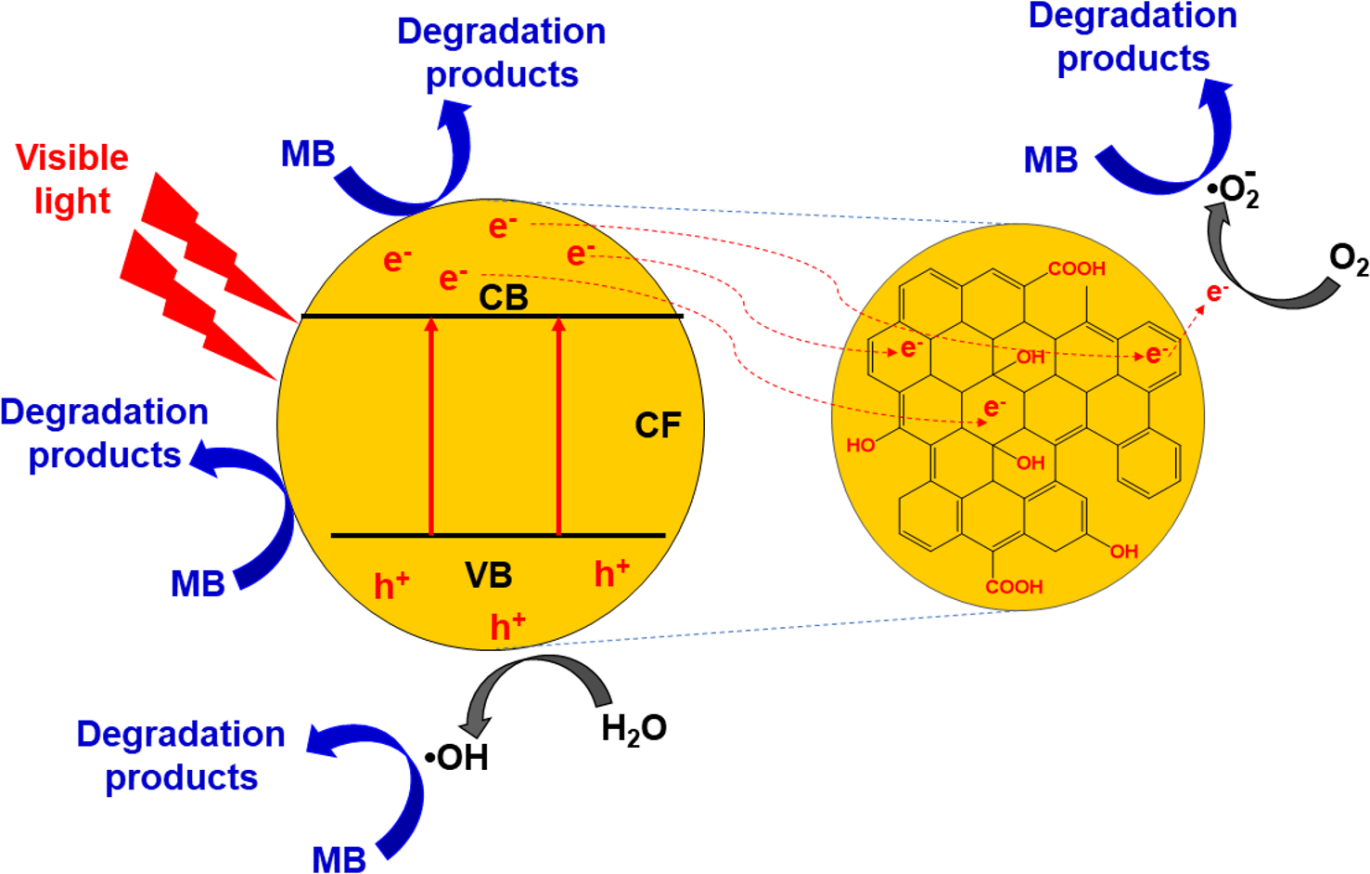
Mechanism of MB degradation over the CF/GQDs catalyst.

The mechanism of MB degradation over the CF/GQDs catalyst is illustrated in Scheme 2. Under visible light irradiation, photogenerated holes (h^+^) are created in the valence bands via the transfer of photogenerated electrons (e^−^) from the valence band to the conduction band (Equation 1). Hence, photoinduced electron transfer possibly takes place from CF to GQDs, which are excellent acceptors [39] (Equation 2). This retards the recombination of electrons and holes in the nanocomposites [40]. The photoinduced holes are, therefore, available to react with H_2_O to produce ^•^OH radicals. The electrons in the CB band directly reduce MB to its degradation products or interact with oxygen in the aqueous solution to form peroxy anion radicals (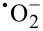). A part of h^+^ oxidizes water to form hydroxyl radicals (^•^OH), or it can oxidize directly MB to its degradation products (Equations 4–6). Finally, the degradation products decompose into CO_2_. The reactions are illustrated as follows:


[1] $$\text{CF}+h\nu \to \text{CF}\left({\text{e}}_{\text{CB}}^{-}+{\text{h}}_{\text{VB}}^{+}\right)$$

[2] $$\text{CF}\left({\text{e}}_{\text{CB}}^{-}\right)\to \text{GQDs}\left({\text{e}}_{\text{CB}}^{-}\right)$$

[3] $$\text{GQDs}\left({\text{e}}_{\text{CB}}^{-}\right)+\text{MB}\to \text{degradation products}$$

[4] hVB++H2O→O⋅−H+H+

[5] O⋅−H+MB→degradation products

[6] $${\text{h}}_{\text{VB}}^{+}+\text{MB}\to \text{degradation products}$$

[7] $$\begin{array}{l} \text{degradation products}+\text{radicals}\left({\text{e}}_{\text{CB}}^{-},{\text{h}}_{\text{VB}}^{+}{,}^{\cdot -}\text{OH}{,}^{\cdot }{\text{O}}_{2}^{-}\right)\\ \to {\text{CO}}_{2}+{\text{H}}_{2}\text{O} \end{array}$$

## Conclusion

CoFe_2_O_4_/GQDs were successfully synthesized in a thermal hydrolysis process from Co(NO_3_)_2_, Fe(NO_3_)_3_, and starch. The CoFe_2_O_4_ nanoparticles of around 20 nm are highly dispersed in GQDs. The CoFe_2_O_4_/GQDs exhibit superparamagnetic properties and are separable by applying an external magnetic field after the reaction. The mineralisation of MB via photocatalytic degradation is complete. The remarkable enhancement of the CoFe_2_O_4_/GQDs is attributed to active sites of GQDs and excellent heterojunctions between CoFe_2_O_4_ and GQDs. The former is favourable for more MB molecules to adsorb on the active sites of the photocatalysts, and the latter can facilitate the GQDs layers acting as electron acceptors, which are beneficial for suppressing the recombination of photogenerated electrons and holes. These results suggest that the CoFe_2_O_4_/GQD nanocomposite is a potential catalyst for methylene blue degradation and various environmental applications.

## Supporting Information

Fragmentation spectrum of MB and HPLC spectrum of MB at initial and photocatalytic degradation after 180 min.

File 1 Detailed information on MS spectra and HPLC diagram.

## Data Availability

The data that supports the findings of this study is available from the corresponding author upon reasonable request.
