# Supplementary material for: Photocatalytic degradation of methylene blue under visible light by cobalt ferrite nanoparticles/graphene quantum dots
Source: Beilstein J Nanotechnol. 2024 Apr 29;15:475–89. doi: 10.3762/bjnano.15.43 (PMC11074707; doi:10.3762/bjnano.15.43)
Supplement: File 1 — Detailed information on MS spectra and HPLC diagram. [file Beilstein_J_Nanotechnol-15-475-s001.pdf]

BEILSTEIN JOURNAL OF NANOTECHNOLOGY

Supporting Information

for

Photocatalytic degradation of methylene blue under visible light by cobalt ferrite nanoparticles/graphene quantum dots

Vo Chau Ngoc Anh, Le Thi Thanh Nhi, Le Thi Kim Dung, Dang Thi Ngoc Hoa, Nguyen Truong Son, Nguyen Thi Thao Uyen, Nguyen Ngoc Uyen Thu, Le Van Thanh Son, Le Trung Hieu, Tran Ngoc Tuyen and Dinh Quang Khieu

Beilstein J. Nanotechnol. **2024,** *15,* 475–489. doi:10.3762/bjnano.15.43

Detailed information on MS spectra and HPLC diagram

License and Terms: This is a supporting information file under the terms of the Creative Commons Attribution License (https://creativecommons.org/licenses/by/4.0). Please note that the reuse, redistribution and reproduction in particular requires that the author(s) and source are credited and that individual graphics may be subject to special legal provisions.
The license is subject to the *Beilstein Journal of Nanotechnology* terms and conditions: (https://www.beilstein-journals.org/bjnano/terms)

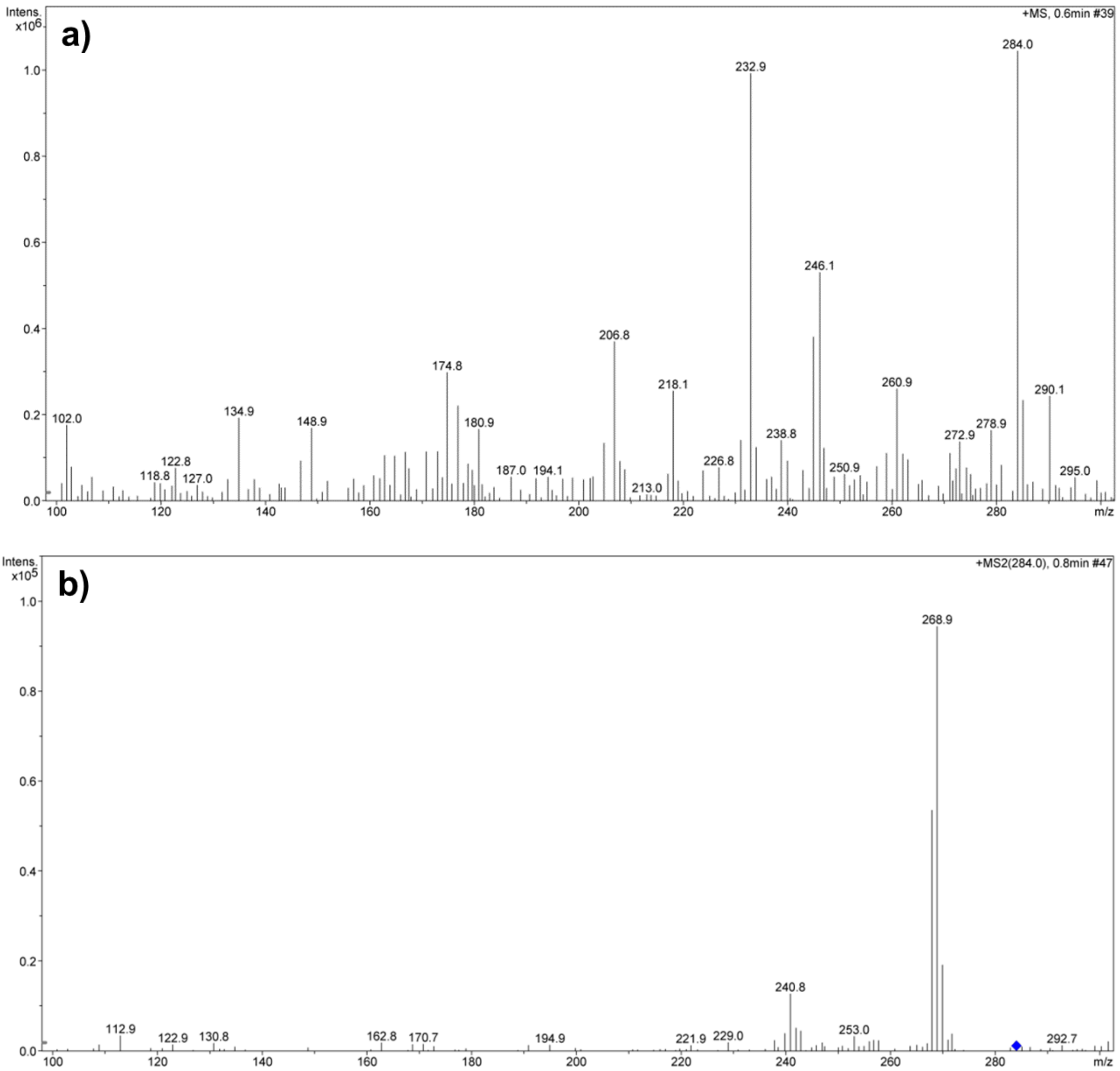

Figure S1: Fracmentation spectrum of MB a) *m*/*z* = 284, b) *m*/*z* = 268.

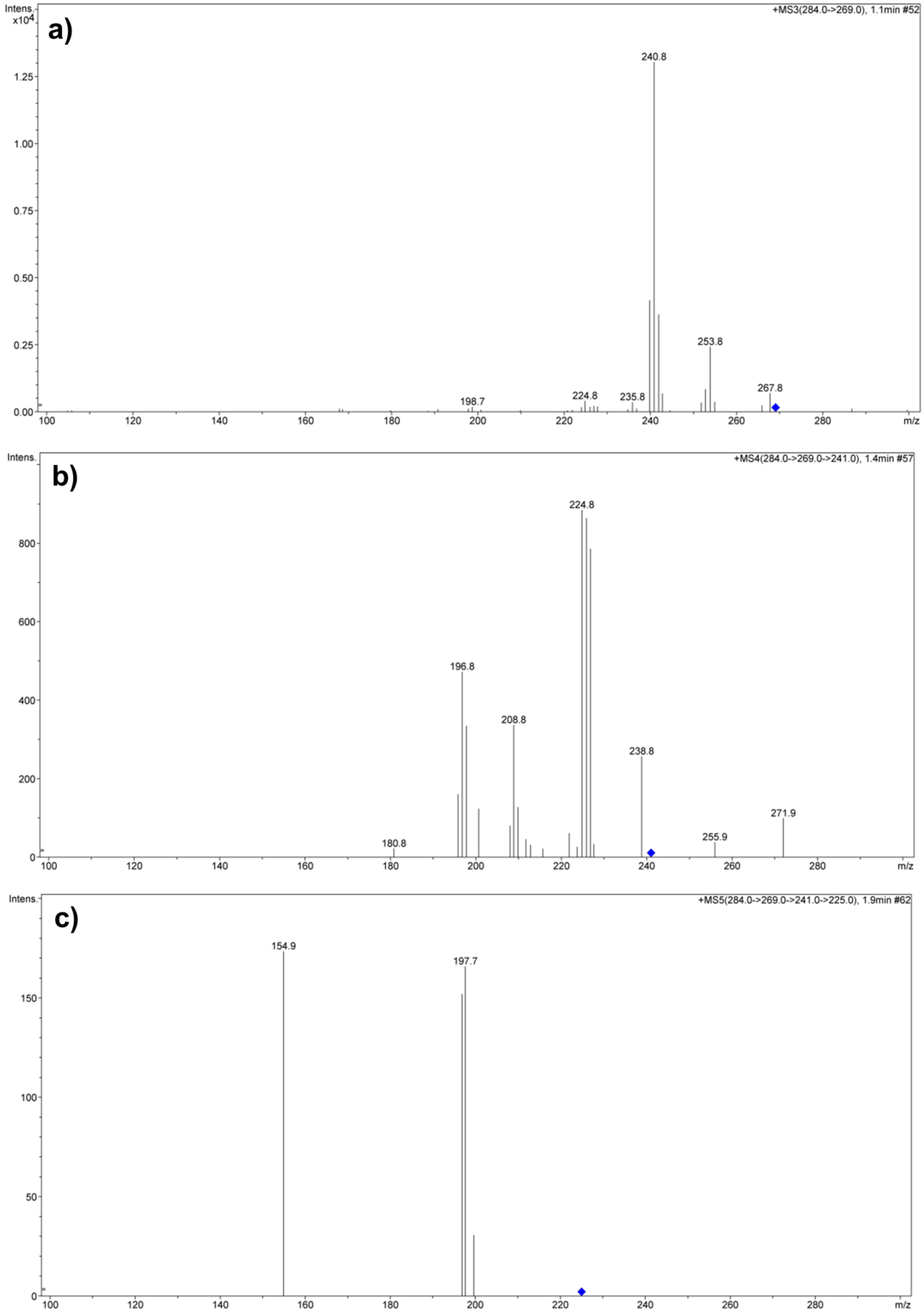

Figure S2: Fracmentation spectrum of MB a) m/z = 240.8, b) m/z = 224.8 and c) m/z = 154.9.

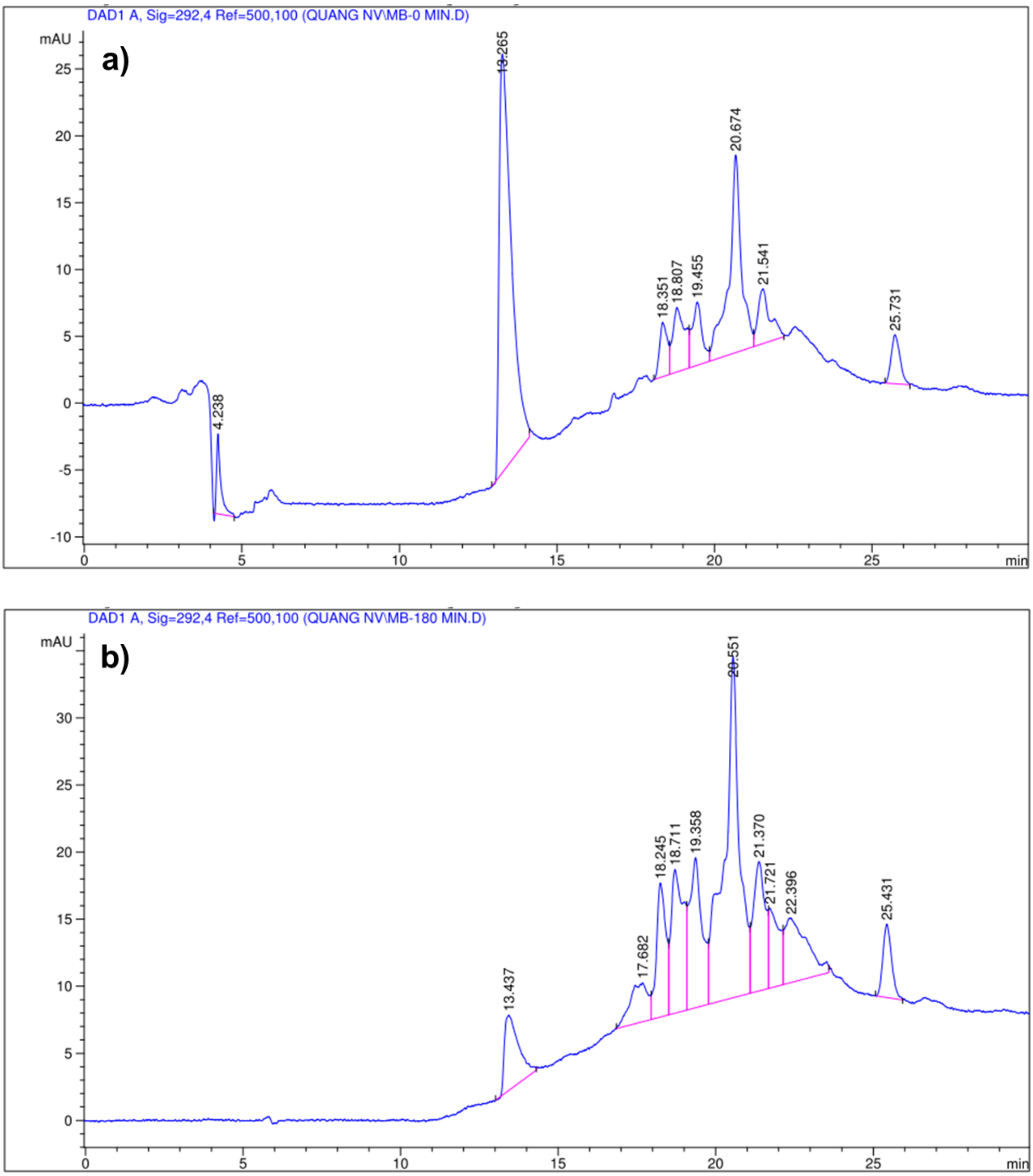

Figure S3: HPLC of MB at a) initial and b) 180 min.
